# System-level analysis of metabolic trade-offs during anaerobic photoheterotrophic growth in *Rhodopseudomonas palustris*

**DOI:** 10.1186/s12859-019-2844-z

**Published:** 2019-05-09

**Authors:** Ali Navid, Yongqin Jiao, Sergio Ernesto Wong, Jennifer Pett-Ridge

**Affiliations:** Physics and Life Sciences Directorate, Lawrence Livermore National Laboratory, 7000 East Ave., Livermore, CA 94550 USA

**Keywords:** Multi-objective analysis, Metabolic trade-offs, Flux balance analysis, Genome-scale model, *Rhodopseudomonas palustris*, Phototrophic metabolism, Autotrophy, Light limitation, Carbon efficiency, Carbon storage

## Abstract

**Background:**

Living organisms need to allocate their limited resources in a manner that optimizes their overall fitness by simultaneously achieving several different biological objectives. Examination of these biological trade-offs can provide invaluable information regarding the biophysical and biochemical bases behind observed cellular phenotypes. A quantitative knowledge of a cell system’s critical objectives is also needed for engineering of cellular metabolism, where there is interest in mitigating the fitness costs that may result from human manipulation.

**Results:**

To study metabolism in photoheterotrophs, we developed and validated a genome-scale model of metabolism in *Rhodopseudomonas palustris*, a metabolically versatile gram-negative purple non-sulfur bacterium capable of growing phototrophically on various carbon sources, including inorganic carbon and aromatic compounds. To quantitatively assess trade-offs among a set of important biological objectives during different metabolic growth modes, we used our new model to conduct an 8-dimensional multi-objective flux analysis of metabolism in *R. palustris*. Our results revealed that phototrophic metabolism in *R. palustris* is light-limited under anaerobic conditions, regardless of the available carbon source. Under photoheterotrophic conditions, *R. palustris* prioritizes the optimization of carbon efficiency, followed by ATP production and biomass production rate, in a Pareto-optimal manner. To achieve maximum carbon fixation, cells appear to divert limited energy resources away from growth and toward CO_2_ fixation, even in the presence of excess reduced carbon. We also found that to achieve the theoretical maximum rate of biomass production, anaerobic metabolism requires import of additional compounds (such as protons) to serve as electron acceptors. Finally, we found that production of hydrogen gas, of potential interest as a candidate biofuel, lowers the cellular growth rates under all circumstances.

**Conclusions:**

Photoheterotrophic metabolism of *R. palustris* is primarily regulated by the amount of light it can absorb and not the availability of carbon. However, despite carbon’s secondary role as a regulating factor, *R. palustris’* metabolism strives for maximum carbon efficiency, even when this increased efficiency leads to slightly lower growth rates.

**Electronic supplementary material:**

The online version of this article (10.1186/s12859-019-2844-z) contains supplementary material, which is available to authorized users.

## Background

The high-throughput “omics” revolution has resulted in a deluge of system-level information about the components of living organisms. Optimally, integration and interpretation of these data can provide mechanistic insights about cellular behaviors and function [1]. This new information can also be used by synthetic biologists to manipulate the biochemical processes within select (primarily microbial) organisms in order to achieve desired outcomes, such as production of valuable compounds like drugs or biofuels. Biofuels generated via microbial metabolism are of significant interest because they could theoretically serve as a primary source of energy for industry and transportation, thus supplanting fossil fuels and mitigating the harmful effects of global climate change.

These positive attributes have resulted in significant interest in conducting system-level analyses of those modes of metabolism that can sustainably result in generation of biofuels, *i.e*, those modes of metabolism that use renewable resources and release part of the generated energy in useful forms. Metabolism in phototrophic organisms, as well as those that can catabolize aromatic compounds (a major component of plant biomass), are such modes of metabolism.

To manipulate cellular metabolism to achieve a desired biological task (or objective), while mitigating the fitness costs that may result from our tampering, it is necessary to have a quantitative knowledge of a system’s critical objectives. This is because it is important to ensure our goals do not significantly alter the natural balance of objectives in a system for a given environment. Life is based on a series of trade-offs that represent the price living organisms pay in term of fitness when improvement in one of their traits results in detrimental change in another; a system state called ‘Pareto efficient’. Evolution ensures that all living organisms are Pareto efficient; otherwise, on an evolutionary timescale the non-efficient would be outcompeted and outlasted by organisms with better performance in all or some biological tasks.

Knowing the nature and magnitude of biological trade-offs can provide invaluable information regarding the biophysical and biochemical bases behind observed cellular phenotypes. This type of knowledge can be gained through the use of genome-scale models (GSM) and system-level multi-objective analyses of cellular processes. Genome-scale mathematical modeling of metabolic networks is a key tool of systems biology that has been used to examine the biochemical underpinnings of cellular phenotypes in microbes ranging from model organisms like *Escherichia coli* [2, 3] and baker’s yeast [4], to those of ecological and industrial interest [5–8], and even deadly pathogens [9, 10]. A number of models have also been developed for photosynthetic organisms, such as purple non-sulfur bacterium *Rhodobacter sphaeroides* [11] and cyanobacterium *Synechococcus* sp. PCC 7002 [12]. When used with constraint-based methods like flux balance analysis (FBA) [13, 14], these models can quantitatively describe the metabolic network’s fluxes under a steady state assumption. This permits analyses of different types of omics data via in silico simulation of all processes of interest following assorted genetic and environmental perturbations [15].

Despite its many uses [15, 16], standard FBA is insufficient for analysis of trade-offs between large numbers of objectives in a system. FBA examines the feasible flux patterns in a system while optimizing a single biological objective function. To examine trade-offs between different objectives of a system, a multi-objective flux analysis (MOFA) approach is needed. MOFA is based on the widely used multi-objective optimization (MO) method, which is a critical tool in a number of fields where a decision maker needs to consider trade-offs between various conflicting objectives. The desired outcomes of MO simulations are Pareto-optimal (PO) solutions. A PO solution of a problem is one for which any improvement in value of one objective will lead to diminishment of another [17, 18]*.*

To date, there have been a number of important MO analyses of biological processes using constraint-based models [19–24]. Phenotype phase plane analysis is one such method used to study the optimal utilization of a system’s metabolic network as a function of variations of two environmental constraints [25, 26]. Thus, this method examines the interactions between three system objectives (growth and the two constraints). Other MO-based studies have provided important insights for bioengineering of systems such as the relationships between environments and regulatory mechanisms [27–29], the minimal number and combination of augmentations to a system that would result in greatest amount of strain optimization [30, 31], and guidelines for tuning synthetic biology devices [32].

All these MO analyses (including MOFA) are based on a reconstruction of the system’s metabolic network. Like FBA, the networks are constrained based on fundamental physico-chemical laws and experimental observations and measurements. For all MO analyses, the *n*-dimensional (*n* = number of objectives) solution space of the model is discretized, and a Pareto optimal solution is calculated for each section of the space. The combined set of these Pareto optimal solutions forms the *n*-dimensional Pareto front for the MO analysis. A variety of different methods are used to identify the examination loci and hence constrain the values for *n*-1 objectives. Subsequently, like FBA, the value of the one remaining objective is optimized.

In this study, we report the first results of a MOFA examination of metabolic trade-offs in a phototrophic organism. The modeled organism, *Rhodopseudomonas palustris* (RP)*,* is a purple non-sulfur (PNS) proteobacterium from the *Rhodospirillaceae* family. RP’s metabolism is extremely versatile and serves as a model for several important biological phenomena, including biodegradation of industrial waste [33, 34], electricity generation [35], and production of hydrogen gas (H_2_) [36–38]. RP has the capability to switch between four different types of metabolism (photoautotrophy, photoheterotrophy, chemoautotrophy, and chemoheterotrophy). It can grow in both aerobic and anaerobic conditions while using light and organic compounds as energy sources, and organic or inorganic [38–40] compounds as electron sources. RP can also fix both carbon dioxide (CO_2_) and nitrogen gas [41, 42].

Finally, RP can metabolize aromatic compounds as a carbon source in a light-dependent fashion under anaerobic conditions (LN). New insights gained through our MO system-level analyses of this type of RP metabolism are important for industrial and environmental reasons, since microbial production of biofuels as well as bioremediation of aromatic pollutants usually occur in low oxygen environments.

To determine carbon and energy fluxes among RP’s different metabolic modes, we developed a GSM of metabolism in RP*.* Although previously a model of central carbon metabolism in RP had been developed [43], that model did not account for a majority of the metabolic reactions in the system and did not use an RP-specific biomass composition. Our curated genome-scale model incorporates most of the metabolic reactions in RP and uses an RP-specific biomass composition. Our model was extensively curated to ensure proton mass balance, because we discovered (as others have also noted [44, 45]), poor accounting of protons can result in erroneous outcomes. For specific details of our model building process, we refer the reader to the Methods section.

Although system-level MOFA analyses of metabolism have expanded analyses of biosystems beyond FBA’s canonical single objective optimization, to date, the maximum number of objective trade-offs that have been simultaneously examined has not exceeded eight [28]. In one of the most detailed analyses, researchers studying trade-offs among a larger group of objectives examined trade-offs among different combinations of 3–5 objectives [46]. While this method would work for analysis of a small set of objectives; when analyzing larger sets of objectives, in order to avoid incomplete considerations of feasible functional capabilities of the system, one would need to analyze an ever-increasing number of small subsets of objectives.

Given RP’s versatile metabolism, our system-level MOFA analysis of its metabolism meant we needed to simultaneously examine trade-offs among more than five interdependent biological objectives and environmental constraints. To this end, we developed a computational algorithm for MOFA that (like another published MO systems biology study [20]) uses the Normalized Normal Constraint (NNC) method [47] to generate multidimensional Pareto solutions for our analyses. In our program, the number of objectives that can be analyzed simultaneously is unlimited. However, as the number of examined objectives increases, the computational resources needed for the calculations increase nonlinearly.

In addition to our MOFA analyses of metabolic objective trade-offs, we used the RP GSM to: a) investigate RP’s metabolism of different carbon sources, b) examine the role of proton availability in affecting mode of metabolism, and c) study RP’s capacity to produce H_2_.

## Results & discussion

First, we used available RP flux measurements [48] for photoheterotrophic metabolism of acetate to validate our model’s predictions. Next, we used acetate import and CO_2_ export flux measurements to constrain our model based on an experimentally observed metabolic phenotype. This allowed us to assess the metabolic limitations of the system. Using this new-found insight and MOFA we then examined the relative importance of different biological objectives during different forms of mixotrophic metabolism. The results of these analyses are detailed below. We also used FBA to examine the robustness of RP’s metabolism to genetic perturbations. The results of these analyses are included in the Additional files 1 and 2.

### Metabolic trade-offs during mixotrophic growth

Recently Shuetz et al. [46] showed that when examining trade-offs between double and triple combinations of different biological objectives in microbes, a PO combination of three tasks – maximum biomass yield, maximum yield of ATP, and optimal allocation of resources – best explains the measured flux distribution for a variety of organisms and conditions. Henceforth we will refer to these objectives as primary objectives. While the primary objectives are the top evolutionarily important objectives and their combined optimization best describes observed metabolic fluxes among all examined trios of objectives, the match with experimental results is not exact. To improve the match between optimization predictions and flux measurements, Pareto optimization of other biological objectives (henceforth labeled secondary objectives) that are pertinent to specific organisms and growth conditions, could help. Examination of secondary objectives also provides us with quantitative insights into how these activities influence cellular workings and could be used to assess the effects of metabolic engineering on the normal workings of a system. The effects of secondary objectives could be particularly pronounced in metabolically versatile organisms. In this study, we examined energy and carbon trade-offs for different types of phototrophic metabolism. So, in order to gain a more complete understanding of the system, besides the three primary objectives, we examined objectives related to environmental nutritional conditions as well as production of compounds of interest (such as H_2_ gas). We examined the RP’s metabolism on 4 different carbon sources (one aliphatic and three aromatic) and the role of proton economy in each case. We also used GX-FBA [49], an in silico method that uses transcriptomic data as constraints for GSMs (see Methods), and permits examination of changes in metabolism of a system as it transitions between various environments and types of carbon sources. The results of these analyses are included in the Additional files 1, 2 and 3.

#### Light-anaerobic metabolism of acetate

**Fig. 1:**
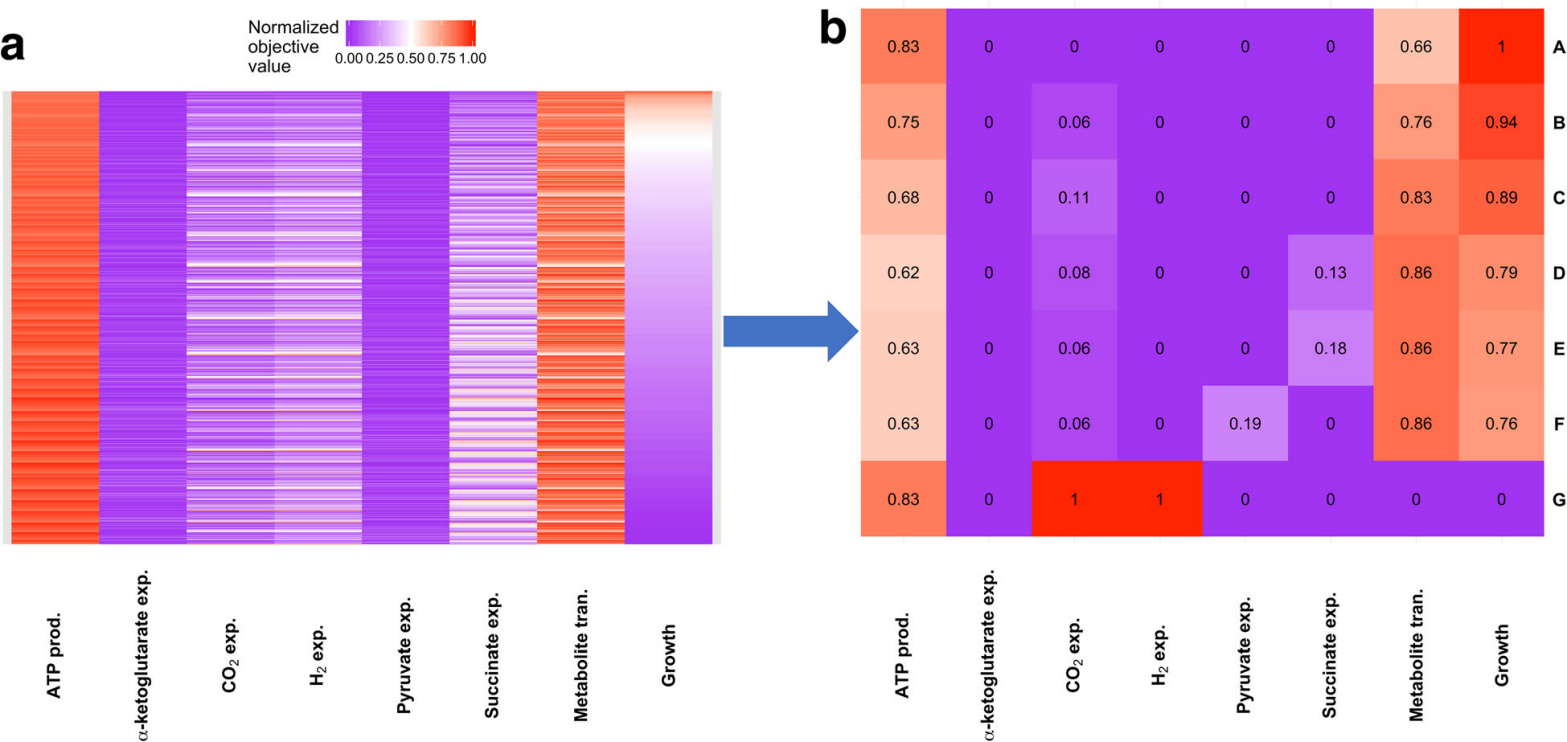
Heat map of the 8-dimensional Pareto front from MOFA analysis (objectives: growth, carbon fixation/carbon efficiency, nutrient allocation, ATP production, production of some small organic byproduct (pyruvate, succinate and α-ketoglutarate), and H_2_ production) of anaerobic mixotrophic metabolism of acetate in *R. palustris*. **a** 1719 unique Pareto-optimal solutions identified during our analysis. **b** Select Pareto-optimal solutions from same MOFA study are highlighted for discussion. Each biological objective was examined at intervals equaling 1/10 maximum normalized value. The analyses showed that the observed growth rate (E) is smaller than the maximum theoretical growth rate (A) in a carbon-limited system with unlimited light absorbing capability. When CO_2_ export was constrained with experimental measurements (without limiting light) the predicted growth rate was still larger than the measured value (B). Eliminating CBB (with unlimited light) resulted in increased production of CO_2_, but the predicted growth rate was still greater than the measured value (C). Limiting light and carbon import brought the predicted growth rate closer to measured value (D), however the rate of CO_2_ export was greater than measured value. When light, acetate import, and CO_2_ export were limited the model predicted that succinate will be exported. Blocking export of succinate slightly lowered the growth rate and resulted in production of other small organic acids (F). Finally, the simulations showed that H_2_ production competes with growth pathways for resources and that maximum theoretical H_2_ production (G) would result in cessation of growth. This result is supported by experimental observations [61]

One of the most detailed studies of metabolism in RP is McKinlay et al.’s examination of photoheterotrophic growth on acetate [48]. The flux measurements reported in that manuscript provided us with a detailed snapshot of the metabolic state of RP during growth in a defined medium, and we used it to quantify the extent to which some biological objectives of RP dictate the behavior of the system under anaerobic mixotrophic conditions. To this end, we conducted a system-level MOFA analysis of LN metabolism of acetate, assuming that the system operated in a Pareto efficient manner (Fig. 1). In order to quantitatively examine how RP allocates its limited resources, we compared the experimental flux measurements with output from an 8-dimensional MOFA study. The examined objectives were: 1) biomass production (growth), 2) CO_2_ export, 3) ATP production, 4) nutrient allocation (minimal metabolite transport), 5) H_2_ export, 6) pyruvate export, 7) succinate export, and 8) α-ketoglutarate export. The latter three objectives were included to examine the role of carbon fixation as a sink for excess electrons. For the nutrient allocation objective, we minimized the sum of the absolute value of the transport rates. In a complex medium, the optimum value of this objective can be degenerate, i.e., different combinations of metabolite imports/exports could result in the same optimum value. However, for our minimal medium, each optimum value results in a singular transport profile.

The growth medium for the MOFA study included acetate as the sole carbon source. It also included ammonium, sulfate, and phosphate as the sole nitrogen, sulfur, and phosphorus sources. Finally, the medium included unlimited photons, as well as Ca^2+^, Cl^−^, and all the metal cations (e.g., Mg^2+^, Mn^2+^, etc.) that are necessary for cellular biomass production.

Figure 1a shows the result of our MOFA analysis. The analysis identifies 1719 Pareto optimal solutions that form the 8-dimensional Pareto front for the examined objectives. As it can be seen, a majority of the Pareto optimal solutions display low growth phenotypes. This is because:

- The majority of the examined objectives have a negative trade-off with respect to growth, i.e., if their values increase then the growth rate reduces.
- The negative effects of these objectives are additive and hence small improvements to the value of all of them will drastically reduce the growth rate of RP.
- Some of imported carbon is exported as reduced organic byproducts, which removes energy needed for biomass formation.

Given the fact the measured growth phenotype is very close to the theoretical growth archetype (a phenotype that optimizes a single task) [50], it can be concluded that a) growth is indeed a primary objective of RP, and b) the secondary objectives that we examined, under normal photoheterotrophic growth conditions, have significantly lower values than their theoretical archetype.

By examining the predicted Pareto optimal phenotypes, we can gain a better understanding of the quantitative trade-offs among the different objectives we examined. Figure 1b highlights some of the Pareto solutions of interest for the photoheterotrophic metabolism of acetate and below we discuss each specific phenotype.

##### Condition 1: Fixed import of acetate

We first used FBA to identify the theoretical growth archetype under carbon-limited conditions. At a fixed uptake rate of acetate that matches experimental flux measurements (1.96 mmol.gDW^−1^.h^−1^), the cell did not produce any small carbon byproducts (Fig. 1b, Row A). This clearly shows that carbon limitation prohibits diversion of resources toward production of non-biomass related byproducts. However, even under this limiting condition, the cell clearly operates near optimum values for the other two primary objectives, i.e., ATP production and resource allocation.

In accordance with the goal of optimizing resource allocation, our results indicate that all the CO_2_ generated from consumption of acetate was fixed and used for production of biomass. For the theoretical growth archetype, FBA flux predictions indicate that the Calvin-Benson-Bassham (CBB) process fixed the majority of the produced CO_2_ (> 70%). Conversion to bicarbonate by carbonate anhydrase (E.C. 4.2.1.1), and pyruvate via pyruvate synthase (E.C. 1.2.7.1) fixed the remaining produced CO_2_. However, the predicted growth rate for the carbon-limited growth archetype was higher than experimentally measured values (predicted doubling time of 6.4 h vs. measured 8.4 ± 0.6 h [51]). This implied that the amount of carbon imported exceeded the growth demands of RP. Thus, we concluded that under the experimental conditions outlined by McKinlay and Harwood [48] for their flux measurements, RP’s metabolism was not carbon limited.

To test the significance of carbon fixation toward maximizing growth, we in silico inactivated CBB (by fixing the reaction rate for RuBisCO to zero). Elimination of CBB increased the predicted doubling time to 7.2 h (still smaller than the measured value) (Fig. 1b, Row C). In the absence of CBB, the predicted rate of CO_2_ export was 21% of the rate of uptake of acetate. This value matches the total measured amount of CO_2_ produced (22% of acetate flux [48]).

Given that the experimentally observed phenotype (Fig. 1b, Row E) includes export of CO_2_, we set the rate of CO_2_ export equal to the measured value (0.23 mmol.gDW^− 1^.h^− 1^). With unlimited light, the predicted doubling time (6.8 h) was still smaller than the measured value (Fig. 1b, Row B). Thus, we found that if light is available and the upper limit for the enzymatic capacity of system to fix carbon has not been reached, CO_2_ will be fixed as long as maximization of growth is the sole objective of the system.

##### Condition 2: Fixed import of acetate, varying absorption of light

We next began examining the effect of light-absorption on the rate of biomass production. Decreasing the amount of light absorbed by the cells increased their doubling time, which improves the agreement between model predictions and experimental observations. However, when the rate of light absorption decreased, the rate of CO_2_ export began to rise above experimentally observed values. To examine what drives these observations:

1. At the measured CO_2_ export rate, we reduced the rate of light absorption until the doubling time matched the experimentally measured value. This was achieved at the photon uptake rate of 36.6 mmol.gDW^− 1^.h^− 1^, suggesting a light-limited metabolism. This is consistent with previous examination of another phototrophic organism [52] and our understanding of RP’s natural ecological niche within light-limited environments (RP grows underneath cyanobacteria in microbial mats [53]). As we decreased the level of absorbed light, the model predicted that RP begins to export succinate (Fig. 1b, Row E; representing 18% of the carbon imported as acetate).
2. When succinate export is blocked, we found a 1% reduction in maximum growth rate, and pyruvate is exported instead (Fig. 1b, Row F). The reduced growth rate is attributable to higher energy cost for the production of pyruvate from acetate in comparison to succinate.

##### Condition 3: Fixed light import, fixed CO_2_ export

At a fixed rate of photon absorption, with no limit to acetate uptake, the model predicted that 17% less acetate consumption results in a slightly (1%) higher growth rate. This result suggest that the measured rate of acetate import is higher than the amount needed for achieving the experimentally measured growth rate.

##### Condition 4: Fixed acetate and light import

Finally, the model showed that at a fixed rate of photon and acetate uptake, if the limit on CO_2_ export is removed, CO_2_ export increases, growth rate increases by ~ 3%, and the rate of small organic acid export drops to ~ 13% of imported carbon (Fig. 1b, Row D). This outcome suggests that the measured CO_2_ export rate is lower than what is needed for achieving the theoretically predicted growth archetype.

The last model prediction implies that the cell uses processes other than export of CO_2_ to expunge excess carbon. Instead, the cells divert a fraction of the absorbed and metabolically generated energy for the production and export of small organic acids, likely driven by redox balance or regulatory constraints.

This result slightly differs from that found in another in silico analysis of redox balancing and biohydrogen production in PNS [43]. Hadicke et al. were able to grow PNS photoheterotrophically by only exporting CO_2_ and biomass [43]. The difference can be explained by the fact that, in our analysis, we constrained the model with three measured values (growth, CO_2_ export rate, and acetate import rate) and allowed for export of small organic compounds. In contrast, Hadicke et al. [43] blocked the export of all byproducts other than CO_2_ and did not fix the rates of light absorption or CO_2_ export. Thus, they were able to predict photoheterotrophic growth with only CO_2_ and biomass as byproducts (similar to our outcome for condition 1), whereas our model with fixed experimental measurements and light absorption, required export of excess imported carbon via means other than CO_2_.

Our 8-dimensional MOFA analysis of LN growth of RP on acetate revealed a pattern consistent with Schuetz et al. [46], i.e., maximizing efficient resource allocation (97% of optimal value), ATP production (84% of maximum value), and growth (the main FBA assumption, 79% of its theoretical maximum value) are the top 3 biological objectives that are optimized. However, our results also seem to indicate that a fourth objective — production of small organic acids (while light energy is available) — is also optimized.

Optimization of this fourth objective diminishes the ability of the cell to achieve the growth archetype for the amount of resources that are imported. Model predictions suggest that the cell by design diverts some of the absorbed light energy toward the production of reduced organic compounds. We can better understand this process by examining the degree of reduction of various carbon-based compounds imported and exported by the system. The degree of reduction for a compound per carbon atom (κ) can be quantified using the concept developed by Roels [54].

We have noted that our predictions indicate that the cell imports extra carbon and electrons in the form of acetate (κ = 4), uses some of this resource for biomass (κ = 4.19) production and then uses light energy to export the remaining electrons as organic compounds like succinate (κ = 3.5) that although more oxidized than acetate are significantly more reduced than CO_2_ (κ = 0). One possible explanation for this behavior could be that the cell stores some of the available energy as easily metabolized compounds for consumption during dark periods, a behavior that has been observed in cyanobacteria [55].

Another point to keep in mind is that the amount of carbon predicted by the model to be used for the production of reduced byproducts is small and well within the range of uncertainties of experimental measurements. Sensitive experimental analyses to identity and quantity the metabolic byproducts of LN metabolism could either validate our MOFA prediction of organic carbon export or support the previous assumption that CO_2_ is the sole byproduct [43].

#### Light-anaerobic metabolism of aromatic compounds

We also conducted a MOFA analysis to examine the metabolic trade-offs of different biological objectives while consuming 3 different aromatic compounds (Table 1). The growth medium for this study blocked import of all carbon sources except the 3 aromatic compounds. The rate of uptake of the 3 compounds was not constrained. Other than the difference in carbon source, the rest of the medium remained the same as for our LN acetate analysis.

**Table 1:** Summary of model predicted characteristics of light anaerobic mixotrophic metabolism of three aromatic compounds. In each case the model predicted doubling time is smaller than the measured value. To achieve the theoretical maximum growth rates, the cell must extensively use rTCA (a carbon inefficient pathway) to fix CO_2_

Aromatic substrate	Predicted minimum doubling time (hours)	Measured doubling time (hours) [62]	Rate of substrate import (mmol/gDW.h)	Percent of imported carbon as CO_2_
4-Coumarate	9	9.4 ± 0.2	0.33	4.6
4HBZ	8.8	12 ± 0.2	0.44	8
Benzoate	8.7	9.3 ± 0.2	0.43	3

**Fig. 2:**
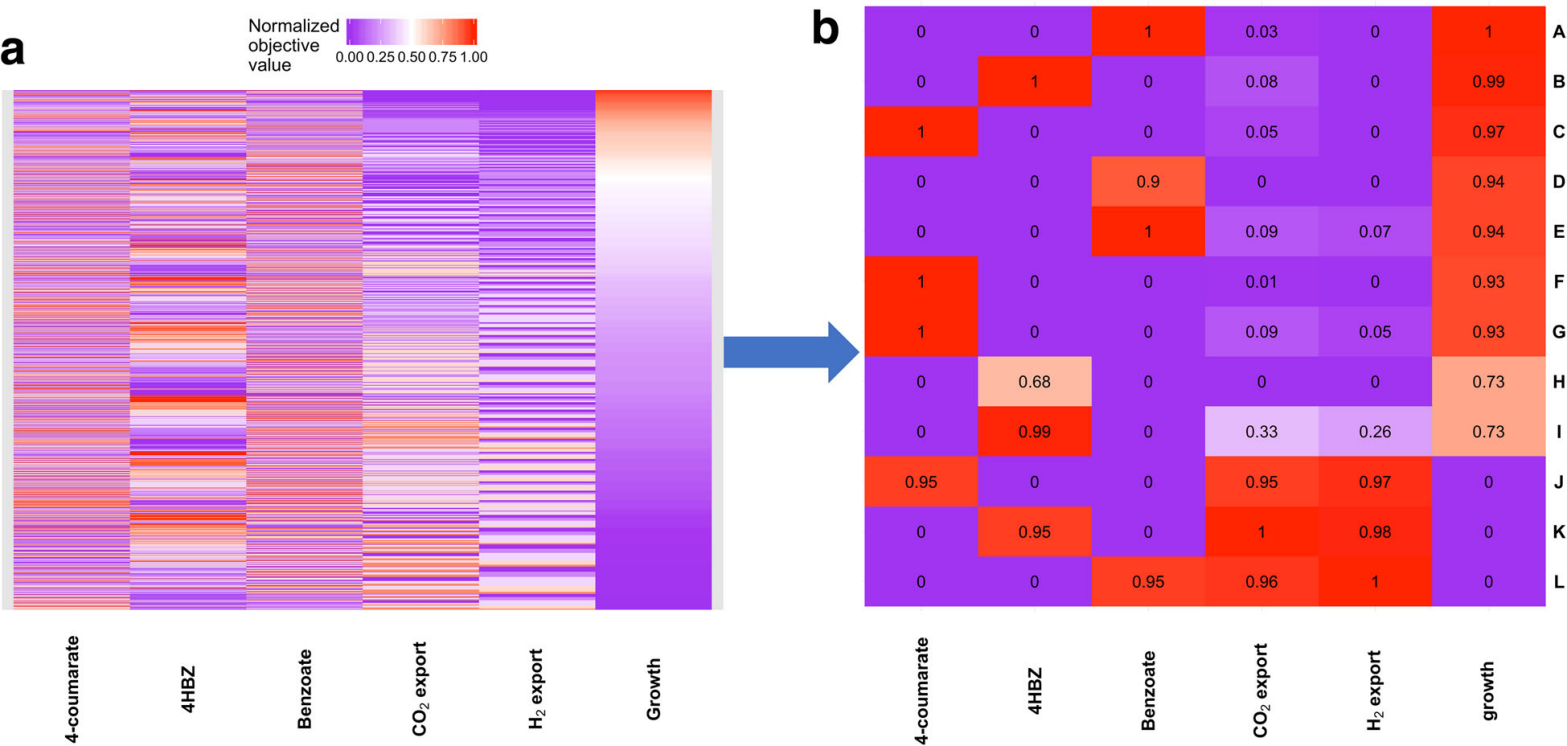
Heat map of the 7-dimensional Pareto front from MOFA analysis of anaerobic mixotrophic metabolism of 3 aromatic compounds in *R. palustris*. **a** 2333 unique Pareto-optimal solutions identified during our analysis. Each biological objective was examined at intervals equaling 1/5 maximum normalized value. **b** Select Pareto-optimal solutions from the MOFA analysis of growth, carbon fixation/carbon efficiency and H_2_ production in RP when growing on a variety of different aromatic compounds. The predicted growth archetypes (A, B & C) have greater growth rates than measured values (D to I). At the measured growth rates, our MOFA analyses predict presence of many metabolic phenotypes. Some (D, F & H) use lower amounts of carbon and fix greater amounts of CO_2_. These phenotypes do not produce H_2_ gas. Others (E, G & I), import extra carbon and use the energy from metabolizing this resource to produce small amounts of H_2_ gas. As with aliphatic metabolism, maximum production of H_2_ results in cessation of growth and full oxidation of the carbon source (J, K & L)

Figure 2a shows the result of our MOFA analysis. The analysis identified 2303 Pareto optimal solutions that form the 7-dimensional Pareto front for the examined objectives. As it can be seen, a significant majority of the Pareto optimal solutions (~ 84%) display growth phenotypes that are less than half that of the growth archetype. This is because:

- Exports of H_2_ and CO_2_ diminish growth. The former siphons electrons and protons needed for growth; while the latter removes carbon that can be fixed back into biomass.
- Excess import of carbon in the absence of absorbing more light or increased export of CO_2_ (a byproduct of chemotrophic metabolism) would lead to export of reduced organic byproducts, which removes energy needed for biomass formation.

The results of our MOFA analyses also indicate that, as with metabolism of acetate, efficient carbon utilization is a primary objective of RP metabolizing aromatic carbon sources. Below we describe the results of our analysis in more detail.

##### Condition 1. Light-anaerobic metabolism of 4-coumarate

Under light-limited anaerobic conditions with 4-coumarate as the sole carbon source, a small fraction (4.6%) of the imported carbon was exported as CO_2_ while the majority of the produced CO_2_ (83%) was incorporated into biomass. Unexpectedly, at the growth archetype (Fig. 2b, Row C), our model predicted that the CBB cycle was not the primary route of carbon fixation. Instead, a large fraction (~ 61%) of CO_2_ was fixed by enzymes pyruvate:ferredoxin oxidoreductase (E.C. 1.2.7.1) and 2-oxoglutarate synthase (E.C. 1.2.7.3). These enzymes are associated with the reductive citric acid cycle (rTCA) which is a pathway for carbon fixation in some photoautotrophic organisms such as the green-sulfur bacterium *Chlorobium limicola* [56] and chemolithoautotrophic archaea [57].

Presence of rTCA in RP is intriguing since it is not present in another well-studied, closely related species, *Rhodobacter sphaeroides*. One advantage of using rTCA for carbon fixation is that it requires less energy than CBB [57]. Thus, given the energy-limited state of the system when light absorption is restricted, using this mode of carbon fixation is essential for achieving the growth archetype.

However, the rTCA-based optimum theoretical growth rate was 4% higher than the measured value (Table 1). At the growth rate that matches experimental measurements (Fig. 2b, Row F), the system can use CBB for carbon fixation (accounting for ~ 56% of the produced CO_2_). The switch to CBB also resulted in a 5% improvement in carbon utilization efficiency. Gene expression analyses have verified extensive use of CBB during LN metabolism of 4-coumarate [58]. Thus, our results indicate that under light-limited conditions RP does not achieve the theoretical growth archetype and instead the primary objective during LN metabolism of 4-coumarate is maximum growth while striving to achieve maximum metabolic efficiency.

##### Condition 2. Light-anaerobic metabolism of benzoate

MOFA analyses of growth archetype with benzoate as the sole carbon source under light-limited anaerobic conditions (Fig. 2b, Row A) predicted that only 3% of the imported carbon was exported as CO_2_ while approximately 90% of the produced CO_2_ was fixed into biomass. However, as with 4-coumarate, due to energy considerations, 67% of CO_2_ was fixed by rTCA. MOFA results at the measured growth rate (Fig. 2b, Row D) showed that the system can switch to CBB for CO_2_ fixation (~ 50% of generated CO_2_). This mode of metabolism is about 2% more carbon efficient than the metabolism at the optimum theoretical growth rate.

##### Condition 3. Light-anaerobic metabolism of 4-hydroxybenzoate

Using the model to examine flux patterns in the growth archetype when RP consumes 4-hydroxybenzoate (4HBZ) under light-limited anaerobic conditions (Fig. 2b, Row B), we found that 8% of the imported carbon was exported as CO_2_. Only about half of the CO_2_ that was produced was fixed through the activity of rTCA while around 20% is fixed through the formation of carbonic acid. The results at the measured growth rate (which was significantly smaller than the predicted growth archetype value, Table 1) identify a likely change in the mode of carbon fixation. At the measured growth rate (Fig. 2b, Row H), the carbon efficient CBB pathway can become the primary route of CO_2_ fixation (83% of produced CO_2_). This mode of metabolism is 8% more carbon efficient than the one used to achieve theoretical optimum growth rate.

Overall, when simulating light-limited (absorption values similar to those calculated for acetate metabolism, 36.6 mmol.gDW^− 1^.h^− 1^) photoheterotrophic growth of RP on aromatic compounds, with exception of metabolism on 4HBZ, the model predicts growth rates that are reasonably close to measured values. This can be viewed as further proof of light-limited nature of RP’s phototrophic metabolism.

Also, if we assume that RP’s enzymatic capacity to fix CO_2_ by rTCA is comparable to that of CBB, then it appears that optimum growth is not the sole objective that controls LN metabolism of aromatics in RP. Our results indicate that the cell grows at the maximum growth rate that also optimizes carbon efficiency. Hence, the cell uses the more energy expensive CBB carbon fixation pathway that results in a lower growth rate in comparison to theoretical growth archetype, but instead minimizes carbon waste.

#### Proton economy of light-anaerobic metabolism

Our simulations of RP acetate metabolism under LN conditions indicate that RP must import protons from the surrounding medium in order to achieve the measured growth rate. This agrees with previous studies that showed exchange of protons with growth medium is important for maximizing cellular growth [2]. It also has been shown that in other species of *Rhodopseudomonas*, lower pH values in the surrounding medium result in an increased rate of biomass production [59].

**Fig. 3:**
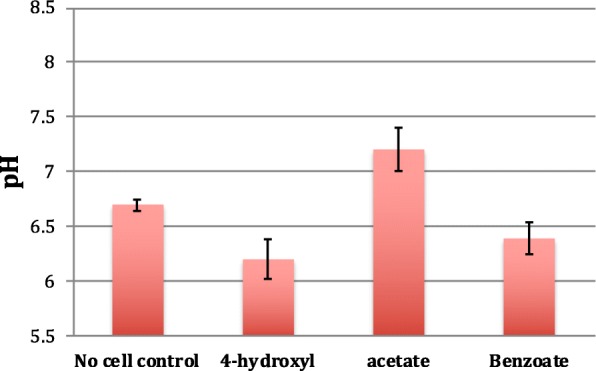
Experimentally measured changes in pH of the growth medium following anaerobic metabolism of 4-hydroxybenzoate, acetate, and benzoate

The imported protons are bound to the excess oxygen atoms that were imported as acetate and are exported as water. The model predicts that without proton uptake and under LN conditions, the growth rate is 13% lower (9.7 h doubling time), due to excess oxygen being exported as α-ketoglutarate, wasting carbon and electrons that could otherwise be used for growth. α-ketoglutarate is exported because it has a low degree of reduction (κ = 3.2). If we eliminate export of α-ketoglutarate, this further reduces the growth rate (10.5 h doubling time) because alternative oxygen carriers (e.g.*,* pyruvate and succinate (κ = 3.5)) contain more reduced carbon than α-ketoglutarate. The model’s prediction that RP imports protons during LN acetate metabolism should lead to a pH increase in the growth media, consistent with our experimental observation. Phototrophic growth of RP on acetate in poorly buffered minimal media leads to a medium pH increase (from 6.7 to 7.2) (Fig. 3).

Examining the proton economy of LN metabolism of aromatic compounds*,* the model predicts that unlike metabolism of acetate, breakdown of some compounds (e.g.*,* benzoate and 4-coumarate) results in production of protons that are exported. This is due to the low (compared to biomass) hydrogen and oxygen content of these aromatic compounds. The reactions for metabolism of 4-coumarate and benzoate are:

1 $$ {\mathrm{CH}}_{0.78}{\mathrm{O}}_{0.33}+0.22\ {{\mathrm{N}\mathrm{H}}_4}^{+}+0.21\ {\mathrm{H}}_2\mathrm{O}\to {\mathrm{CH}}_{1.93}{\mathrm{O}}_{0.54}{\mathrm{N}}_{0.22}+0.15\ {\mathrm{H}}^{+} $$

2 $$ {\mathrm{CH}}_{0.71}{\mathrm{O}}_{0.29}+0.22\ {{\mathrm{N}\mathrm{H}}_4}^{+}+0.25\ {\mathrm{H}}_2\mathrm{O}\to {\mathrm{CH}}_{1.93}{\mathrm{O}}_{0.54}{\mathrm{N}}_{0.22}+0.16\ {\mathrm{H}}^{+} $$

Each carbon that is imported into the cell carries fewer protons and oxygen atoms than acetate (CH_1.5_O) and thus water needs to be used to make up for this shortcoming. Our experiments verified that LN metabolism of benzoate indeed reduces the pH of the growth medium (Fig. 3).

Under light limited conditions, at the measured growth rate, the model predicts that metabolism of 4HBZ should result in import of protons from the medium. This is because the amount of water imported satisfies the oxygen difference between 4HBZ and biomass but not the hydrogen difference. The 4HBZ metabolism equation is:

3 $$ {\mathrm{CH}}_{0.71}{\mathrm{O}}_{0.43}+0.22\ {{\mathrm{N}\mathrm{H}}_4}^{+}+0.11\ {\mathrm{H}}_2\mathrm{O}+0.12\ {\mathrm{H}}^{+}\to {\mathrm{CH}}_{1.93}{\mathrm{O}}_{0.54}{\mathrm{N}}_{0.22} $$

However, our experiments revealed that LN metabolism of 4HBZ reduces the pH of the medium (Fig. 3). We attribute this discrepancy between the model’s predictions and experimental measurements to the fact that the model-predicted metabolism utilizes the theoretical minimum amount of carbon necessary to achieve the growth archetype. If the 4HBZ metabolism of RP is any less carbon efficient than the model prediction, then the proton metabolism of the system would change. This is because production and export of CO_2_ would increase the amount of H_2_O that needs to be imported to maintain the elemental balance of oxygen. Breakdown of water would result in greater production of protons. For example, if as measured for acetate metabolism [48], 10% of the imported carbon is exported as CO_2_, the metabolic process becomes proton producing and the metabolic equation becomes:

4 $$ {\mathrm{CH}}_{0.71}{\mathrm{O}}_{0.43}+0.2\ {{\mathrm{N}\mathrm{H}}_4}^{+}+0.26\ {\mathrm{H}}_2\mathrm{O}\to 0.9\ {\mathrm{CH}}_{1.93}{\mathrm{O}}_{0.54}{\mathrm{N}}_{0.22}+0.1\ {\mathrm{CO}}_2+0.29\ {\mathrm{H}}^{+} $$

Yet again, as with acetate, upon analyzing the experimental observations with the model, it can be construed that the behavior of the cell does not solely optimize just a single biological objective such as growth or maximum nutrient use efficiency, but rather a combination of multiple objectives.

#### Hydrogen gas production by *R. palustris*

Examining hydrogen gas production from *R. palustris’* LN metabolism of acetate, our model predicted a maximum H_2_ production yield of 4 mol H_2_/mole acetate, matching the previously published value [43]. The model predictions also suggest that at both theoretical maximum and observed growth rates, RP should not produce H_2_ gas (Fig. 1b, Rows A and E), in agreement with previous experimental observations [48]. MOFA results showed that H_2_ production negatively effects RP’s growth rate and carbon efficiency (i.e.*,* carbon fixation via the CBB pathway) (Fig. 1b, Row G). Production of small amounts of H_2_ is necessary for the production of small organic acids like pyruvate and α-ketoglutarate. Only CO_2_ production is positively affected by H_2_ production. In solely carbon limited conditions, maximum theoretical H_2_ production requires that RP fully oxidize acetate to CO_2_ and use all the energy generated from this chemical process as well as photon absorption to produce H_2_ gas (Fig. 1b, Row G).

It is known that production of highly reduced energy and carbon storage compounds such a polyhydroxybutyrate (PHB) can negatively affect H_2_ production in PNS [60]. PHB production requires use of significant quantities of reducing equivalents and are needed for H_2_ production. Our simulations concur with these findings. Simulating nitrogen starvation while growing on acetate, our model predicts that RP will produce the subunits (C_4_H_6_O_2_) of PHB, if this compound is allowed to be exported as a metabolic byproduct. When we optimized PHB production while minimizing the total exchange of nutrients and byproducts (at fixed rates of carbon and photon import), RP produced water, PHB (1 PHB/6 acetate), and succinate.

Previous studies have also shown that nitrogen starved, non-growing RP cells produce H_2_ gas along with PHB and α-ketoglutarate and CO_2_ as metabolic byproducts [61]. To test whether we could predict the same metabolic phenotype for the nitrogen-starved and non-growing condition, we blocked export of succinate. This constraint resulted in export of α-ketoglutarate as a byproduct. We also observed that while the total amount of PHB produced was lower than when succinate was exuded as a byproduct, the efficiency of PHB production increased (1 PHB/5 acetate). Thus, it appears that nitrogen-starved RP cells simultaneously maximize PHB production and carbon efficiency while minimizing transport fluxes.

MOFA analyses of H_2_ production during LN metabolism of three aromatic compounds indicate that regardless of carbon source, at the theoretical growth archetypes, RP would not produce H_2_ gas (Fig. 2b, A, B, C). Although the examined aromatic compounds (average κ = 4.17) are more reduced than acetate (κ = 4), and usually carry extra protons (Eqs. 1 & 2); at the growth archetypes, the available energy and reducing equivalents are used to fix CO_2_. At the experimentally measured growth rates, which are lower than the predicted growth archetype rates, the cells can use the excess energy that is not used for production of biomass to produce H_2_.

Production of H_2_ can be induced if the objective of RP’s metabolism is changed from maximized carbon efficiency to one where extra carbon is imported and wasted as CO_2_. For example, when growing on 4-coumarate at the measured growth rate, the model predicts that RP can produce 1.1 mol of H_2_ for every mole of 4-coumarate metabolized (Fig. 2b, Row G). This number is similar to that for benzoate (1.14 H_2_/benzoate) (Fig. 2b, Row E), both of which are significantly smaller than the ratio for 4HBZ (4.6 H_2_/4HBZ, Fig. 2b, Row I). This significantly higher predicted ability to produce H_2_ while metabolizing 4HBZ is due to the fact that the measured growth rate for 4HBZ is ~ 25% slower than for benzoate and 4-coumarate [62]. Thus, in theory, to produce 5 molecules of H_2_ per 4HBZ, the cell imports 45% more carbon and does not use the extra energy that is available from absorbing light to fix CO_2_ via CBB. The available excess energy is instead used to produce H_2_ gas. As with acetate metabolism, for maximum H_2_ production, MOFA analyses predicted absolute cessation of growth (Fig. 2b, Rows J, K, L).

When we examine H_2_ production via phototrophic metabolism of RP, our results consistently show that production of H_2_ diminishes the activity of other important biological objectives that have been considered for FBA analysis, such as optimum growth or metabolic efficiency (Figs. 1 and 2). This is consistent with previous suggestions that H_2_ production competes with biomass generation for resources such as energy, protons, and electrons [61, 63]. So, while H_2_ production can serve as an electron sink similar to CBB; the important difference between the two processes is that the latter conserves cellular resources while the former (due to absence of uptake hydrogenase activity [64] in RP) wastes it.

It appears that expression of nitrogenase automatically results in H_2_ production as long as the redox state of the system provides it with reducing agents [61, 65]. To lower the negative cost of nitrogenase activity, RP regulates nitrogenase expression through nitrogen sensing and post-translational modification [66].

Thus, we surmised that presence of nitrogenase under normal conditions is extremely deleterious to cellular growth as well as a number of other cellular objectives. The enzyme’s main function is to fix nitrogen when this resource is scarce, and given the importance of this task, we hypothesize that it has a very high affinity for the substrates it needs for accomplishing its task, namely reduced ferredoxin and protons. Indeed, computational analyses have shown that the active site of the nitrogenase enzyme has more affinity for protons and electrons than the platinum-based catalysts that are used for abiotic production of H_2_ [67]. Hence, if nitrogenase is expressed, irrespective of the primary objective of the cellular metabolism, it will siphon reduced ferredoxin and protons from other important metabolic processes and, depending on the redox state of the system, produce H_2_. However, these resources are essential for a variety of other important functions. This could explain why layers of transcriptional control (e.g., nifA [68]) and post-translational regulation exist to tightly control nitrogenase activity.

## Conclusions

Our MOFA analyses described herein examined the trade-offs between 8 biological objectives for RP’s photoheterotrophic metabolism of acetate (matching the maximum number of objective trade-offs that have been simultaneously examined to date [28]) and 7 objectives for photoheterotrophic metabolism of aromatic compounds. Our results provide new insights into the phototrophic metabolism of RP and indicate that the rate of light absorption limits cellular growth. While our analyses have shown that like other forms of metabolism [50], the primary objectives that the system optimizes are growth, ATP production, and metabolic efficiency; our results suggest that RP’s phototrophic metabolism is energy limited which defines the order of importance of these objectives. Our results indicate that during LN phototrophic metabolism in RP, optimum allocation of resources and production of ATP are more important than growth. Our results also hint at a preference for a fourth cellular objective during phototrophic growth, i.e., production and excretion of reduced carbon compounds that might be used as an energy source during dark periods.

Our examination of differences between RP’s metabolism of aromatic and aliphatic carbon sources using GX-FBA (see Additional files 1, 2 and 3) indicated that transitioning from the former to latter will result in a reduction of CBB activity, consistent with measurements that suggest downregulation of genes associated with this pathway [58]. We (and others [58]) attribute this to the fact that the generally aromatic compounds are more reduced than aliphatic ones and hence during metabolism of aromatics there is a greater need for use of CBB as an electron sink.

Finally, we found that proton metabolism plays a key role in shaping observed growth phenotypes. Under anaerobic conditions, the ratio of carbon, oxygen and hydrogen in RP’s carbon source relative to its biomass, and the overall carbon efficiency of phototrophic metabolism, determine whether the system prefers a more basic or acidic medium for growth.

## Methods

### Metabolic network reconstruction

The metabolic network reconstruction for *Rhodopseudomonas palustris* (model iAN1128) is based on the annotated genome of *Rhodopseudomonas palustris* CGA009 [41]. Of the 4836 predicted genes present in the genome, 1514 are related to cellular metabolism and biosynthesis. Our model accounts for the activity of 1128 of these genes (75%), resulting in 1000 enzymatic reactions. Additional literature surveys identified the activity of 37 local orphan enzymes (13-critical for biomass production, 20-based on literature, 4-pathway hole-filling) and 14 non-enzymatic reactions, resulting in a final model of 1037 reactions and 949 metabolites.

The initial foundation of the model is a draft model obtained from Model SEED [69]. So, all the initial gene-protein-reaction (GPR) associations were based on RAST annotation [70]. During the curation process, all RAST-based GPR associations were compared against the original annotation [41] as deposited on National Center for Biotechnology Information (NCBI) [71], and KEGG [72]. In cases where there were disagreements between the annotations, the original annotation was used as the highest authority followed by KEGG, and lastly RAST. We prioritized KEGG assignments of function because KEGG ortholog assignment bases are continually updated in the KEGG Orthology And Links Annotation program (KOALA) [72]. Also, we recently showed [73] that when comparing accuracy of enzymatic annotation (characterized by Enzyme Commission (EC) numbers), KEGG predictions are significantly more accurate than RAST for *E. coli*.

The same curation scheme was used to ensure that multiple genes associated with a reaction are correctly labeled as either isozymes or components of an enzyme complex. In cases where multiple reactions were combined into one reaction (e.g., multi-step isomerization reactions), the reaction in the GPR was treated as if it was catalyzed by a multi active-site enzyme complex.

In some cases, we used KEGG to improve/update the originally published annotations. For example, byproducts of some genes in the original annotation have been assigned a clearly annotated function (e.g., phosphoglycerate kinase for the gene RPA0943) but have not been assigned an EC number. In these cases, we used KEGG to assign the correct EC number for the enzyme and the associated reactions were added to the metabolic network reconstruction. In some other cases, the EC numbers assigned in the original annotation have become obsolete. For example, the original annotation assigns the EC number 1.3.99.1 to the byproduct of gene RPA0216. That EC number is no longer in use and has been changed to 1.3.5.1. For such cases we used KEGG to identify and correct these dated assignments and added the correct reactions to our model.

Finally, in some cases KEGG had assigned a function to a protein that did not have one in the original annotation. We did not blindly accept KEGG’s assignment, and instead examined the initial experimental basis for the KEGG ortholog (KO) assignment and verified the KEGG association using BLAST.

In cases where the roles of essential regulatory genes were known, such as the need for hbaR (RPA0673) and aadR (RPA4234) for growth with 4-hydroxybenzoate as the carbon source [74, 75], these associations were incorporated in the model’s GPR basis. But for situations where deletion of a gene reduces RP’s growth rate only under specific media conditions (e.g.*,* badR (RPA0655) mutants grow slowly on benzoate [76]), then the gene was not included in the GPR.

The biomass equation for RP was developed using a variety of data sources. The overall breakdown of cellular components is from McKinlay and Harwood [48]. The amino acid, nucleotide, cofactors, carotenoids and phospholipid composition of the biomass are unique to RP. It has been shown that the composition of RP’s cellular membrane changes when the cell transitions between dark-aerobic environments to LN environments [77, 78]. We implemented this change in our model by developing two separate biomass compositions, with bacteriochlorophyll composition of LN biomass drawn from Firsow et al. [79] and composition of lipids and fatty acids (both dark and light conditions) drawn from Wood et al. [77]. The composition of the polysaccharide moiety of lipopolysaccharides is from Weckesser and Drews [80]. Although in most photosynthetic organisms, genes for carotenoid biosynthesis are simultaneously expressed with other genes involved in chlorophyll biosynthesis and light harvesting process [81], and overall carotenoid concentrations greatly increase between dark and light conditions [82], we did not remove carotenoids from the biomass composition in dark aerobic conditions. This is because carotenoids have other functions in dark conditions, such as quenching free radicals and roles in overall cellular response to environmental stress [83].

It is known that oxygen is not required for the oxidative reactions that are involved in biosynthesis of carotenoids, different forms of quinones, nicotinates, and nicotinamides [84–86]. However, the enzymes associated with these anaerobic transformations are not known. In our RP model, anoxygenic reactions for production of these compounds were drawn from the Model SEED database [69], and were used as orphan reactions without any GPR association.

One significant challenge encountered during the course of our RP GSM development was the unique structure of RP’s Lipid A. The lipid A base of lipopolysaccharides (LPS) in RP has been shown to be composed of 2,3-diamino-2,3-dideoxyglucose [87]; however, the metabolic pathway for production of this compound is unknown. We used microarray analyses to measure the expression of genes known to be associated with usual pathways of LPS production to test whether common pathways for glucosamine-based lipid A synthesis were active in RP. Our analyses showed that most of these genes were prominently expressed in RP. Given this information, we used a number of in silico methods such as AS2TS [88] protein structure modeling tool and tools for identification of catalytic sites [89] and protein function predictions (CATSID) [90] to assess whether any of these enzymes could catalyze production of diamino-glucose. However, based on our analyses of RP proteins, we could not find any enzyme able to catalyze the required chemical reactions.

Our model’s biomass has an elemental composition of CH_1.93_O_0.54_N_0.22_. This formula is somewhat different from that measured for the elemental composition of RP strain 42OL (CH_1.8_O_0.38_N_0.18_) [91]. However, the model’s biomass is closer in composition and degree of reduction per carbon mole (κ = 4.19) to the “standard” biomass formula of CH_1.8_O_0.5_N_0.2_ [92] (κ = 4.2) than the composition for strain 42OL (κ = 4.5). Hence for our simulations the overall formula for conversion of acetate to biomass is:

5 $$ {\mathrm{CH}}_{1.5}\mathrm{O}+0.05\ {\mathrm{H}}^{+}+0.17\ {{\mathrm{N}\mathrm{H}}_4}^{+}\to 0.76\ {\mathrm{CH}}_{1.93}{\mathrm{O}}_{0.54}{\mathrm{N}}_{0.22}+0.29\ {\mathrm{H}}_2\mathrm{O}+0.06\ {\mathrm{CO}}_2+0.18\ \mathrm{CHO} $$

We set the value for non-growth associated maintenance ATP usage to that previously used for *Escherichia coli* (7.6 mmol.gDW^− 1^.h^− 1^) [93]. Variation of this value does not change the outcome of metabolic simulations since changing the rate of light absorption will account for any increase or decrease in this value.

We curated the model extensively to ensure absolute mass balance, including proper proton balance under physiological pH values. We also imposed the loop law on the model and eliminated all thermodynamically infeasible type III extreme pathways [94].

An SBML file of the model is included in the Additional file 3. The model can be also downloaded from bbs.llnl.gov/AliNavid.html and EMBL-EBI’s Biomodels Database [95].

Comparing the predicted metabolic phenotypes with experimental observations validated the model. We examined the model’s ability to consume a variety of different carbon sources as reported in the literature [39, 96]. It is known that strains of RP can consume a large array of different aromatic compounds [97–103]. However, while the mechanism for breakdown of the common intermediate in the process of anoxic aromatic catabolism (i.e., benzoyl-coa) [74, 104–109] has been extensively examined, the enzymatic process and associated genes for breakdown of some parent compounds are not known. Furthermore, strain CGA009 cannot consume some of the aromatic compounds that other strains catabolize. For example, while strain CGA009 cannot use 3-chlorobenzoate [110], RP strain RCB100 uses this compound as a carbon source [111]. Thus, our model only accounts for metabolisms of aromatic compounds whose degradation pathways have been identified (benzoate, 4-hydroxybenzoate, phenol, cresol, coumarate, protocatechoate, vanillate, phenol, and cinnamate).

### Flux balance analysis

The FBA modeling approach uses a genome-scale metabolic reconstruction as its basis. The reconstruction is developed using elementary functional information derived from annotated genomes and available knowledge of enzymology. The reconstruction of an organism’s metabolic reactions is represented as a stoichiometric matrix, *S (m × n)*, where *m* is the number of metabolites and *n* the number of different reactions. Applying the assumptions of mass balance and metabolic steady-state, the following set of linear equations govern the system’s behavior:

$$ \frac{d{X}_i}{dt}=\sum \limits_j{S}_{ij}{\nu}_j=0, $$

where *X*_*i*_ is the concentration of metabolite *i*. For FBA, other limitations are imposed on the system based on experimental studies, including a limit on the amount of flux that courses through a reaction, as well as constraints on the amount of nutrients imported, and the waste products secreted from the system. These constraints are formulated as:

$$ \alpha \le {\nu}_i\le \beta, $$

$$ \chi \le {b}_i\le \varphi, $$

where *b*_*i*_ and ν_*i*_ are the export/import flux of metabolite species *i*, and the flux through internal reaction *i* respectively, and *α*, *β*, *χ*, and *φ* are the lower and upper limits for these fluxes. Finally, FBA utilizes linear programming to determine a feasible steady-state flux vector that optimizes an objective function, most commonly chosen to be the production of biomass, i.e. cellular growth. FBA was used with our RP GSM to analyze single gene knockout phenotypes for all the genes in the model. Several reviews [112, 113] provide detailed description of this process*.*

### GX-FBA

In order to assess differences in RP metabolism when growing on aliphatic and aromatic carbon sources, we used the GX-FBA modeling methodology [49] with available gene-expression measurements [58] for RP growing on different carbon sources. We combined mRNA expression data with a constraint-based framework using the multi-step approach previously detailed for GX-FBA [49]. Note that, for our analyses we chose to only take into account gene-expression changes of at least 50% (±0.5 fold change).

A brief description of GX-FBA steps is:

1. Generate the flux distribution ${\nu}_i^1$ for the starting condition (1) using an Interior Point optimization algorithm with biomass growth or any other appropriate goal as the objective function.
2. For nutritional constraints associated with the post-transition environment (condition (2)), flux variability analysis (FVA) [114] with minimal flux for biomass production set to zero is utilized to calculate the lower and upper fluxes that each model reaction *i* (${v}_i^{\mathrm{min}}$ and ${v}_i^{\mathrm{max}}$ respectively) can carry solely based on environmental limitations and network connectivity. From these results, the mean possible flux value for each reaction (${\overline{v}}_i$) and average flux carried by all active reactions (${\overline{v}}^{\mathrm{all}}$) is determined.
3. Identify the set of reactions *T* for which an mRNA expression value can be associated. For protein complexes and reactions catalyzed by isozymes, the maximal up- or down-regulation value is used unless the mRNA expression values are inconsistent (mixture of up- and down-regulation). In the latter case, the reaction is excluded from *T*.
4. Construct a new objective function:

$$ Z=\sum \limits_{i\in T}{\log}_2\left({C}_i^{mRNA}\right)\frac{\nu_i}{{\overline{\nu}}_i}. $$

If the flux value for condition 1 of a reaction *i* is zero, ${\nu}_i^1$ and ${\overline{v}}_i$ are set equal to the average value for all active reactions (${\overline{v}}^{\mathrm{all}}$). For a more detailed description of this method see Navid and Almaas (2012) [49].

### Catalytic site identification server

The catalytic site identification (CATSID) web server [89, 90] rapidly identifies structural matches to a user-specified catalytic site among all Protein Data Bank proteins. It also examines a user-specified protein structure or model to identify structural matches to a library of catalytic sites. CATSID includes a database of pre-calculated matches between all Protein Data Bank proteins and the library of catalytic sites. The databank has been used to derive a set of theorized new enzymatic function annotations. Matches and predicted binding sites can be visualized interactively online. We used CATSID along with a number of other in silico methods for examining protein structure such as AS2TS [88] to determine if whether any of the enzymes encoded by RP genome could catalyze production of diamino-glucose, a key subunit of RP Lipid A.

### Multi-objective flux analysis

As with the effort by Nagrath et al. [20], our MOFA program uses the Normalized Normal Constraint (NNC) method [47] to map the *n*-dimensional Pareto front of the competing metabolic objectives. NNC generates an even distribution of Pareto solutions on convex or non-convex Pareto frontiers for problems of *n*-objectives. Additionally, NNC is usable for an arbitrary number of objectives and its results are entirely independent of the scales of the examined objectives scales.

The mathematical representation of a generic multi-objective optimization problem is:

$$ \underset{x}{\min}\left\{{z}_1(x),{z}_2(x),\dots, {z}_n(x)\right\},\left(n\ge 2\right) $$

subject to:

6 $$ {g}_j(x)\le 0,\left(1\le j\le r\right) $$

7 $$ {h}_k(x)=0,\left(1\le k\le s\right) $$

8 $$ {x}_l\le {x}_i\le {x}_u,\left(1\le i\le {n}_x\right) $$

Vector *x* denotes the design variables (fluxes) and *z*_*n*_ denotes the *n*th objective function. Equations 6–8 denote the inequality, equality and side constraints.

NNC can be briefly described as a method where an investigator’s choice of a set of *n* objectives defines an *n*-dimensional volume in which all Pareto solutions to the problem are found. Next, *n* anchor points are identified. Anchor Points are feasible solutions, in the objective space, that correspond to the best possible values for respective individual objectives. The values of the anchor points are normalized to eliminate deficiencies associated with scales of individual objectives. The solution space volume is then reduced through the use of an *n*-dimensional “Utopia” hyperplane. The Utopia plane is defined such that it contains all *n* anchor points. Finally, a set of evenly distributed points on the Utopia hyperplane serve to constrain the all but one of the objectives under consideration. Solving for the optimal value of the lone objective at each one of these points will result in calculation of a Pareto solution. For a full mathematical description of NNC see the manuscripts by Dr. Achille Messac and coworkers [47, 115].

It is interesting to note that Shoval et al. [50] recently showed that best trade-off phenotypes for any organism are the weighted averages of archetypes. In the NNC method anchor points represent these archetypes. Results from Shoval et al. also indicate that experimentally observed phenotypes are contained within simple geometric shapes that are akin to the Utopia line, plane, or hyper-plane (depending on the dimension of MO analysis) in NNC – i.e., the geometric space defined by the anchor points.

For our MOFA analyses, we used the RP GSM and changed the constraints based on the growth medium we were examining. Based on the choice of the *n* objectives we were examining; the algorithm would determine the appropriate *n* anchor points to define the Utopia plane for the problem. At each point on the hyperplane, the value of *n*-1 objectives would be constrained, and then (like FBA) linear programming will be used to solve for the optimum value of the remaining objective.

### Analysis of growth-related pH changes in the medium

*Rhodopseudomonas palustris* CGA009 was grown in modified photosynthetic medium [116] with low phosphate under anaerobic conditions. To observe how pH of the growth media was affected by bacterial growth on various carbon sources, we lowered the phosphate concentration to 20% (5 mM) of the original concentration and adjusted the initial pH to 6.7 prior to cell inoculation. An organic source of acetate (10 mM), benzoate (3 mM), 4-hydroxylbenzoate (2.2 mM) or 4-coumarate (2 mM) was provided as the sole carbon source. Anaerobic cultures were placed 20 cm away from a 60 W incandescent light bulb under constant light, and optical density (OD at 660 nM) was monitored to calculate doubling time. The pH of the spent media was measured upon cultures reaching late exponential phase. Three biological replicates were included for each condition.

## Additional files

Additional file 1: System-level Analyses of robustness of metabolism to environmental and genetic perturbations. (DOCX 3179 kb)

Additional file 2: RP model and gene knockout results. (XLSX 133 kb)

Additional file 3: SBML format of the RP model. (XML 1614 kb)

## Abbreviations

4HBZ: 4-hydroxybenzoate
CBB: Calvin-Benson-Bassham
FBA: Flux balance analysis
GSM: Genome-scale model
LN: Light, anaerobic condition
MO: Multi-objective
MOFA: Multi-objective flux analysis
NNC: Normalized normal constraint
PHB: Polyhydroxybutyrate
PNS: Purple non-sulfur
PO: Pareto-optimal
RP: *Rhodopseudomonas palustris*
rTCA: Reductive citric acid cycle
