# Supplementary material for: System-level analysis of metabolic trade-offs during anaerobic photoheterotrophic growth in Rhodopseudomonas palustris
Source: BMC Bioinformatics. 2019 May 9;20:233. doi: 10.1186/s12859-019-2844-z (PMC6509789; doi:10.1186/s12859-019-2844-z)
Supplement: Supplementary file 1 — System-level Analyses of robustness of metabolism to environmental and genetic perturbations. (DOCX 3179 kb) [file 12859_2019_2844_MOESM1_ESM.docx]

Supplementary Material for “System-level analysis of metabolic trade-offs during anaerobic photoheterotrophic growth in *Rhodopseudomonas palustris*”

## ***System-level* Analyses of robustness of metabolism to environmental and genetic perturbations**

**Metabolism of aliphatic compounds**

One of the most important uses of constraint-based analysis of GSMs has been to assess the robustness of the organism of interest’s metabolism to genetic and environmental perturbations. Using FBA, we conducted a series of *in silico* single reaction (SRKO) and gene knockout (SGKO) analyses of RP metabolic network. Table S1 lists the essential reactions and genes required for growth under different carbon source, light and oxygen availability scenarios.

When comparing robustness of RP acetate metabolism (as the sole carbon source) in dark aerobic (DA) and light anaerobic (LN) conditions, our SGKO and SRKO analyses of the system show that in DA environments, the system is more robust to metabolic network perturbations (334 essential reactions and 252 essential genes—about 9% fewer essential genes). Examining the LN succinate metabolism, the number of essential genes (284) and reactions (359) are similar to those for acetate. The only differences are that succinate dehydrogenase is essential for growth on succinate; while aconitase is essential when acetate is the sole carbon source.

***Metabolism of aromatic compounds***

For over two decades, scientists have extensively studied RP’s LN metabolism of aromatic compounds and have painstakingly identified the enzymes involved in this process. The LN metabolism on a diverse array of aromatic compounds proceeds through a limited number of intermediates that are amenable to the reduction of the aromatic ring in the absence of oxygen. The most important intermediate in RP’s LN metabolism of aromatic compounds is benyzol-CoA[1]. The “peripheral” pathways that convert various growth substrates to benzoyl-CoA include a variety of different classes of reactions such as carboxylation of phenolic compounds, oxidation of methyl substituents, reductive elimination of ring substituents (e.g., hydroxyl or amino groups), and shortening of aliphatic side chains (see Figure S1). Following these preparatory steps, benzoyl-CoA is metabolized to acetyl-coA through the “central” portion of anaerobic aromatic metabolism. This process involves a biological Birch-like[2] reduction of the aromatic ring followed by ring cleavage and ultimately a β-oxidation[3].

Our system-level robustness analyses of LN phototrophic metabolism of aromatic compounds show that RP lacks alternatives to the central breakdown pathway. A majority of the steps shown in Figure S1 can be inactivated through SGKOs. However, the “central” pathway is more robust to mutations than the “peripheral” pathways. Of the 13 steps involved in the process of converting benzoyl-CoA to acetyl-CoA, only 5 are susceptible to inactivation through SGKOs.

The model only includes the non-β-oxidation route for conversion of 4-coumaroyl-CoA to 4-hydroxybenzoyl-CoA (see Figure S1). Although there exists a possibility for a β-oxidative pathway, gene-expression studies[4] have provided strong support for the non-β-oxidative pathway in RP. The first step of the non-β-oxidative pathway involves the removal of an acetyl-CoA from 4-coumaroyl-CoA by an enoyl-CoA hydratase/lyase to produce 4-hydroxybenzldehyde (see Figure S1). Because this step removes a fraction of the imported 4-coumarate and transforms it into acetyl-CoA that can easily be metabolized by the system, our *in silico* SGKO analyses for growth on 4-coumarate indicate that, unlike growth on benzoate or 4-hydroxybenzoate (4HBZ), the genes involved in breakdown of benzoyl-CoA are high fitness cost but not essential for growth with 4-coumarate. When genes associated with benzoyl-CoA metabolism are deleted, the system is expected to export 4HBZ as a byproduct, with growth rate slowing down by 77%. Although export of 4HBZ during metabolism of 4-coumarate has been observed[5], the non-essentiality of enzymes involved in metabolism of 4HBZ and benzyol-CoA has not been experimentally verified.

Analyses of robustness of LN aromatic metabolism of RP to genetic mutations predict a number of false positives. For example, the model predicts that PimCD enzymes (RPA3713 and RPA3714) are essential for breakdown of the important aromatic intermediate benzoyl-coA due to their role catalyzing reduction of pimeloyl-CoA (E.C. 1.3.1.62). However, RP must have other enzymes that can catalyze this step because it has been shown that deletion of the pim operon in RP does not stop cellular growth but only reduces the growth rate when metabolizing aromatic compounds in comparison to wild type strains[6]. Without knowing the identity of these isozymes, it is not possible to eliminate predictions of false-positive essential genes.

| Carbon source | # of essential reactions | # of essential genes |
| --- | --- | --- |
| Acetate  Acetate*  Succinate  4-hydroxybenzoate  4-benzoate  4-coumarate | 357  334  354  368  367  356 | 280  252  281  300  294  283 |

Table S1. Results of in silico analyses of reaction and gene essentiality for LN metabolism of different carbon sources in Rhodopseudomonas palustris (*=dark aerobic).

***Examination of differences between RP’s metabolism of aliphatic and aromatic carbon sources***

Our result for aliphatic metabolism of RP, and previous analyses[7], have highlighted the importance of carbon fixation to achieving the growth archetype in phototrophs like RP. Interestingly, transcriptomic measurements suggest that RP upregulates genes associated with CBB after switching from growth on an aliphatic carbon source (succinate) to aromatic compounds like 4-coumarate or benzoate[4]. Given our prediction that CBB is the primary pathway of carbon fixation during aliphatic metabolism of RP, the increased use of CBB for aromatic metabolism warranted further investigation. To this end, we used GX-FBA[8], an *in silico* method that uses transcriptomic data as constraints for GSMs (see methods), and permits examination of changes in metabolism of a system as it transitions between various environments.

We used the transcriptomic data[4] to explore RP’s metabolism as it switches from aromatic to aliphatic carbon sources. Figure S2 illustrates the change of metabolic pathway fluxes as RP’s carbon source shifted from 4-coumarate to succinate. GX-FBA predicts that the transition results in a reduction in activity of CBB which is consistent with measured downregulation of genes associated with this pathway[4]. We (and others[4]) attribute this to the fact that the examined aromatic compounds are more reduced (κ>4.1) than succinate (κ=3.5) and hence need greater use of CBB as an electron sink. In addition, GX-FBA predicted that the switch to the aliphatic carbon source results in an increase in fluxes through pathways for metabolism of cysteine (κ=5.67), methionine (κ=6), and pyruvate metabolism. Given the highly reduced nature of these sulfur-based amino acids, it is curious that a switch from the more reduced aromatic to less reduced aliphatic carbon source results in increased production of these compounds. These seems to indicate that there is a reason beyond redox balance associated with these metabolic changes. The aromatic to aliphatic change in carbon source also results in a decrease in fatty acid (κ>4.67) metabolism, metabolism of lysine (κ=4.67) and tryptophan (κ=4.18), and activity of the benzoate degradation pathway. Transition from benzoate to succinate resulted in a similar pattern. The reduced activity of the benzoate pathway would be expected and could be directly linked to reduced production of the aromatic amino acid phenylalanine. Reduced availability of electron following the switch could explain why production of highly reduced fatty acid compounds is predicted.


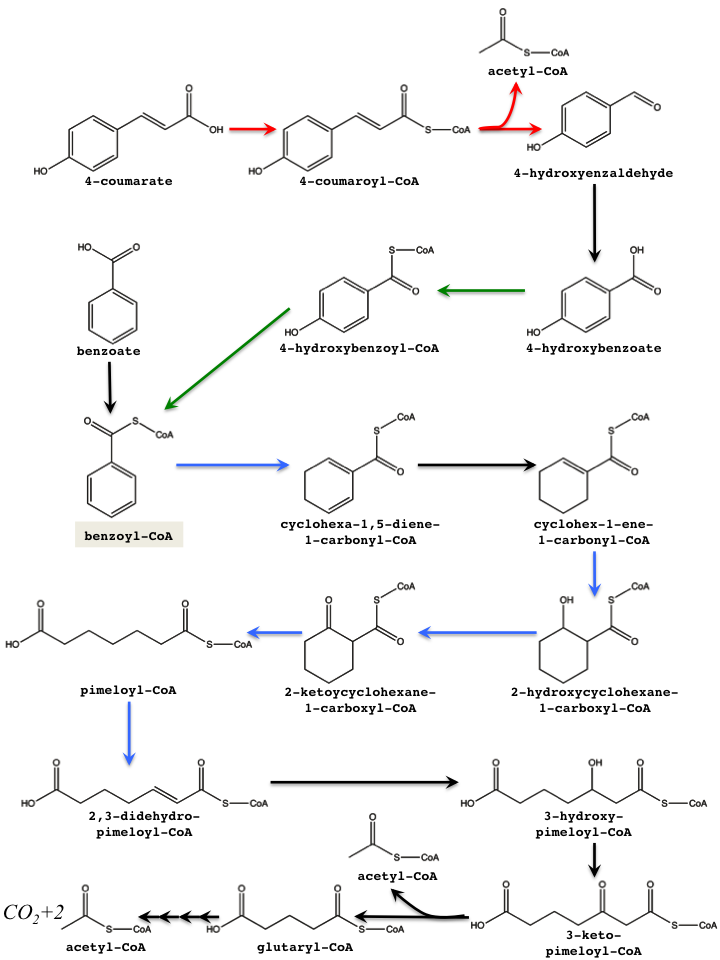


Figure S1. Diagram showing the pathway for anaerobic metabolism of various aromatic compounds. During metabolism of 4-coumarate, a unit of acetyl-CoA is produced prior to production of benzoyl-CoA. Thus, growth on 4-coumarte is more robust to genetic perturbations than growth on other common aromatic compounds like benzoate and 4-hydroxybenzoate. Red arrows highlight steps in metabolism of 4-coumarte that are susceptible to SGKOs. Green arrows identify steps that are susceptible to SGKOs and are uniquely essential for metabolism of 4HBZ. Blue arrows represent metabolic steps that are susceptible to SGKOs for metabolism of both benzoate and 4HBZ.

Figure S2. GX-FBA predicted change in metabolic pathway activity in *Rhodopseudomonas palustris* after changing the carbon source from 4-coumarate to succinate. The transition leads to reduced carbon fixation via CBB. Blue=flux decrease, red=flux increase, green=flux did not increase or decrease by at least a factor of 2. The graph is made using the iPath2 program[9] and the width of the lines (w) is set to: $w=20+\log_{10} \left( \frac{v_{i}^{succ}}{v_{i}^{4Coum}} \right)$. If the calculated w<0 for sake of being able to notice the change w=1.

References:

1. Hutber GN, Ribbons DW: **Involvement of coenzyme A esters in the metabolism of benzoate and cyclohexanecarboxylate by Rhodopseudomonas palustris**. *Journal of general microbiology* 1983, **129**(8):2413-2420.

2. Birch AJ: **117. Reduction by dissolving metals. Part I**. *Journal of the Chemical Society (Resumed)* 1944:430-436.

3. Harwood CS, Burchhardt G, Herrmann H, Fuchs G: **Anaerobic metabolism of aromatic compounds via the benzoyl-CoA pathway**. *FEMS Microbiology Reviews* 1999, **22**(5):439-458.

4. Pan C, Oda Y, Lankford PK, Zhang B, Samatova NF, Pelletier DA, Harwood CS, Hettich RL: **Characterization of Anaerobic Catabolism of p-Coumarate in Rhodopseudomonas palustris by Integrating Transcriptomics and Quantitative Proteomics**. *Molecular & Cellular Proteomics* 2008, **7**(5):938-948.

5. Harrison FH: **Peripheral pathways of anaerobic benzoate degradation in Rhodopseudomonas palustris**. University of Iowa; 2005.

6. Harrison FH, Harwood CS: **The pimFABCDE operon from Rhodopseudomonas palustris mediates dicarboxylic acid degradation and participates in anaerobic benzoate degradation**. *Microbiology* 2005, **151**(3):727-736.

7. Hädicke O, Grammel H, Klamt S: **Metabolic network modeling of redox balancing and biohydrogen production in purple nonsulfur bacteria**. *BMC systems biology* 2011, **5**(1):150.

8. Navid A, Almaas E: **Genome-level transcription data of Yersinia pestis analyzed with a New metabolic constraint-based approach**. *BMC Syst Biol* 2012, **6**(1):150.

9. Yamada T, Letunic I, Okuda S, Kanehisa M, Bork P: **iPath2. 0: interactive pathway explorer**. *Nucleic acids research* 2011, **39**(suppl 2):W412-W415.
